# Repositioning FDA-Approved Sulfonamide-Based Drugs as Potential Carbonic Anhydrase Inhibitors in *Trypanosoma cruzi*: Virtual Screening and In Vitro Studies

**DOI:** 10.3390/ph18050669

**Published:** 2025-05-01

**Authors:** Eyra Ortiz-Pérez, Adriana Moreno-Rodríguez, Timoteo Delgado-Maldonado, Jessica L. Ortega-Balleza, Alonzo González-González, Alma D. Paz-González, Karina Vázquez, Guadalupe Avalos-Navarro, Simone Giovannuzzi, Claudiu T. Supuran, Gildardo Rivera

**Affiliations:** 1 Laboratorio de Biotecnología Farmacéutica, Centro de Biotecnología Genómica, Instituto Politécnico Nacional, Reynosa 88710, Mexico; eortizp@ipn.mx (E.O.-P.); titi_999@live.com (T.D.-M.); jessica_ortega7@hotmail.com (J.L.O.-B.); al.gonzalez.gonzalez88@gmail.com (A.G.-G.); apazg@ipn.mx (A.D.P.-G.); 2 Laboratorio de Estudios Epidemiológicos, Clínicos, Diseños Experimentales e Investigación, Facultad de Ciencias Químicas, Universidad Autónoma “Benito Juárez” de Oaxaca, Avenida Universidad S/N, Ex Hacienda Cinco Señores, Oaxaca 68120, Mexico; arimor10@hotmail.com; 3 Facultad de Medicina Veterinaria y Zootecnia, Universidad Autónoma de Nuevo León, General Escobedo 66050, Mexico; kwvazque@gmail.com; 4 Departamento de Ciencias Médicas y de la Vida, Centro Universitario de la Ciénega (CUCIÉNEGA), Universidad de Guadalajara, Av. Universidad 1115, Lindavista, Ocotlán 47820, Mexico; guadalupe.avalos5337@academicos.udg.mx; 5 Neurofarba Department, Section of Pharmaceutical Sciences, University of Florence, Via Ugo Schiff 6, Sesto Fiorentino, 50019 Florence, Italy; simone.giovannuzzi@unifi.it (S.G.); claudiu.supuran@unifi.it (C.T.S.)

**Keywords:** *Trypanosoma cruzi*, carbonic anhydrase, drug repurposing, virtual screening, molecular docking

## Abstract

**Background/Objectives:** α-carbonic anhydrase (α-TcCA) has emerged as a promising drug target in *T. cruzi*, the causative agent of Chagas disease in the Americas. Sulfonamides, known inhibitors of CAs, bind to the zinc ion on the enzyme’s active site. This study proposes the repositioning of sulfonamide-based drugs to identify new trypanocidal agents. **Method:** Ligand-based virtual screening and molecular docking analysis were performed on FDA-approved drugs targeting α-TcCA. These compounds were evaluated in vitro and ex vivo against the A1 and NINOA strains, followed by enzymatic assays. **Results:** Four sulfonylureas were selected: glimepiride (Glim), acetohexamide (Ace), gliclazide (Glic), and tolbutamide (Tol). Ace and Tol had half-maximal inhibitory concentration (IC_50_) values similar or better than reference drugs against the NINOA strain in the epimastigote and trypomastigote stages, while Glic and Glim had the highest activity against the A1 strain (epimastigotes and amastigotes). Notably, Ace had the highest trypanocidal activity against all stages in NINOA, with IC_50_ values of 6.5, 46.5, and 46 μM for epimastigotes, trypomastigotes, and amastigotes, respectively. Additionally, Ace inhibited α-TcCA with K_I_ = 5.6 μM, suggesting that its trypanocidal effect is associated to the enzyme inhibition. **Conclusions:** This study supports the repositioning of FDA-approved sulfonamide-based hypoglycaemic agents as trypanocidal compounds. Future studies should focus on structural modifications to improve selectivity. Integrating docking, parasitological, and enzymatic data is crucial for optimizing drug candidates for Chagas disease.

## 1. Introduction

American trypanosomiasis or Chagas disease, caused by the parasite *Trypanosoma cruzi* is considered one of the most serious parasitic diseases in the Americas. Due to limited and scarce pharmacological treatment, it has been included in the World Health Organization’s list of fourteen neglected diseases [1]. Beyond its primary host and vector (the triatomine bug), this parasite has been found in more than 100 mammalian reservoir species, particularly those involved in the domestic and peri-domestic cycle [2]. The life cycle of this parasite is well known and consists of several morphologically distinct stages: blood trypomastigotes and amastigotes in humans; metacyclic trypomastigotes and epimastigotes in the vector [3]. The disease is divided into two phases: acute and chronic. In the acute phase, symptoms are very mild, and patients may suffer from headache, dizziness, fever, hepatic and splenomegaly. In the latter, the parasite load decreases, and the disease remains clinically asymptomatic for years (indeterminate phase) while the parasite slowly replicates in the tissues, creating nests of amastigotes that cause cardiac, digestive, or neurological changes that can lead to death. Unfortunately, most cases are diagnosed in the chronic stage, when the disease has caused irreversible damage and there is little that can be done [4,5].

Benznidazole (Bzn) and Nifurtimox (Nfx) (1966–1970) were approved to treat this disease; however, both drugs have significant disadvantages such as limited efficacy, toxicity, and resistance [6], leading to a search for new therapeutic alternatives [7,8,9].

Over time, several therapeutic targets have been explored for the development of new trypanocidal drugs, including ergosterol [10], the triose phosphate isomerase (TIM) pathway [11,12], *trans*-sialidase [13], cruzain [14], dihydrofolate reductase-thymidylate synthetase (DHFR-TS) [15], and recently, carbonic anhydrase (*CA*). Carbonic anhydrases are metalloenzymes found in all living organisms and are responsible for catalyzing a simple but fundamental reaction: CO_2_ + H_2_O ⇌ H_2_CO_3_ ⇌ HCO_3_^−^ + H^+^. In *T. cruzi*, a unique isoform belonging to the α-family with similar kinetic parameters of catalytic activity has recently been characterized (k_cat_ = 1.21 × 10^6^ s^−1^, K_m_ = 8.1 × 10^−3^ M k_cat_/K_m_ = 1.49 × 10^8^ M^−1^ × s^−1^) and compared to the most important human isoform II (k_cat_ = 1.40 × 10^6^ s^−1^, K_m_ = 9.3 × 10^−3^ M k_cat_/K_m_ = 1.50 × 10^8^ M^−1^ × s^−1^), suggesting that this enzyme plays a crucial role in the parasite’s life cycle [16].

The study of the mechanism of action of one of the most selective inhibitor groups against CAs began in 1940, when the scientists Mann and Keilin [17] reported the selective inhibition of CAs by sulfonamides and their derivatives, leading to the discovery of essential antihypertensive [18,19], diuretic [20], antiglaucoma [21], and antithyroid [22] agents and, in the last two decades, sulfonamides have been under investigation as new agents in chronic and emerging diseases, e.g., in cancer [23,24,25] and parasite research [26]. However, using cost and time-efficient strategies, the large library of sulfonamide-based CA inhibitors can be repurposed for new pharmacological indications. Drug repurposing significantly reduces risk and development costs by providing experimental, safety, absorption, distribution, metabolism, excretion, clinical, and biological data. This has resulted in significant savings, as the time-to-cost ratio for drug repurposing is approximately $300 million/6 years [27], compared to the estimated cost of drug discovery using traditional methods of >$2 billion/10–15 years [28]. Therefore, in this study, we conducted a virtual screening based on molecular docking of FDA-approved sulfonamide-derived drugs aiming to evaluate their potential inhibitory activity against the α-TcCA enzyme. The most promising candidates were subsequently assessed across three developmental stages of *T. cruzi* using two Mexican strains (A1 and NINOA) and enzymatic assays. This approach allowed us to identify FDA-approved sulfonamide-based compounds with potential trypanocidal activity for drug repurposing.

## 2. Results

### 2.1. Virtual Screening

Initially, 11,585 available drugs from the DrugBank database (https://go.drugbank.com/ accessed on 26 June 2022) were analyzed and filtered in DataWarrior to remove duplicates and select only sulfonamide derivatives with the methanesulfonamide moiety (CH_5_NO_2_S) as a free group or embedded in the chemical structure. After that, 635 drugs (Supplementary Materials, Table S1) were analyzed by molecular docking on the active site of α-TcCA. A total of 203 drugs passed the cut-off (−6.9 kcal/mol) set by the control drug acetazolamide (DB00819_Aaz); however, drugs classified by the platform as experimental or investigational were excluded. Forty-five drugs with the best binding free energy (BFE) from −6.9 to −8.9 kcal/mol (Table S2) were obtained, which are classified as diuretics, anti-inflammatories, antihypertensives, antibacterials, antidepressants, and hypoglycaemics, among others (Table 1).

**Figure 1:**
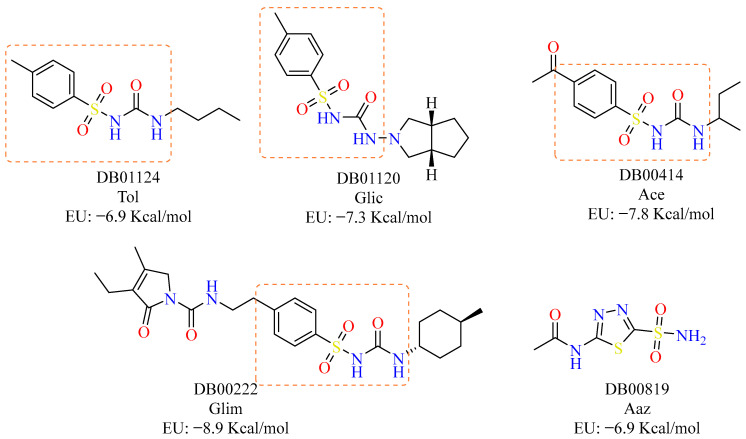
Drugs selected from molecular docking with gnina 1.0 for evaluation. Acetazolamide (Aaz) was used as a control inhibitor [29].

Analysis of the drug candidates showed that DB00222 (Glim), with a binding energy of −8.9 kcal/mol, topped the list; this drug has been used since 1995 as a member of the second generation of sulfonylureas in the treatment of type 2 diabetes mellitus. In contrast, DB01124 (Tol), with a binding energy of −6.9 kcal/mol, also used in the treatment of type 2 diabetes mellitus, was found in last place. Based on these data, in this work, we set out to evaluate, in an in vitro and ex vivo model of *T. cruzi,* the “highest” and the “lowest” drug in terms of molecular binding (kcal/mol), as well as two drugs with intermediate values also belonging to the hypoglycaemic group, Glic, and Ace, with −7.26 and −7.75 kcal/mol, respectively (Figure 1). The 4-methylbenzenesulfonamide scaffold characteristic of these drugs is shown in the orange box.

### 2.2. Interaction Profile Analysis

The active site contains three conserved residues: H94, H96, and H119 (according to the nomenclature of human isoform II). These residues coordinate the metal cofactor, forming the catalytic triad. Additionally, glutamic acid (E106) and threonine (T199) are involved in substrate orientation during catalysis [30]. These are equivalent to H158, H160, H177, E164, and T256 in the AF-Q4CVY4-F1 model of *T. cruzi* (Figure S1).

Interaction analysis revealed that 75% of the forty-five drugs interacted with T256 via hydrogen bonding (HB) and hydrophobic interactions (HI); 71% with H158 via π-cation (π-c), HB, π-stacking (π-s), and salt bridge (SB); 24% with the cofactor (Zn); 20% with H160 via HB; and only 7% with H177 (Figure S3). The molecular docking used in this work confirmed that Aaz interacted with Zn, T256 (HB), and T257 (HB), in addition to H158 (π-c), V193 (HI), and T120 (HB), but there was no interaction with E106 (Figure S2).

**Figure 2:**
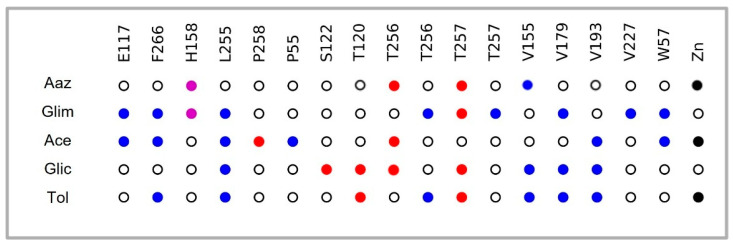
Interaction profile of four selected drugs on the active site residues (mainly with T256 and T257) of α-TcCA and cofactor Zn. Importantly, an interaction pattern was shown with residues such as V155, V179, L255, and F266 (Figure S2), as well as the rest of the drugs resulting from the screening (Figure S3). Blue: Hydrophobic interactions; purple: π-cation; red: hydrogen bond; black: metallic bond; white: no interaction.

As far as the four selected drugs are concerned, Glim (DB00222), Ace (DB00414), and Tol (DB01124) interacted with T256 via hydrogen bridge (HB) and hydrophobic (HI) bonds; this residue has been reported to be important for its crucial role in orienting the substrate for catalysis. Glim (DB00222) also had a π-cation (π-c) interaction with H158, one of the histidine that is part of the catalytic triad. Ace and Tol had an interaction pattern directly with the Zn cofactor (Figure 2).

### 2.3. Trypanocidal Activity

Four hypoglycaemic drugs were evaluated against the three stages in the NINOA and A1 strains, as well as against J774.2 mouse macrophage cells. The results are summarized in Table 2. Glic and Glim had high trypanocidal activity against the A1 strain, particularly in the epimastigote stage, with IC_50_ values of 10.7 μM and 37.6 μM, respectively, surpassing at least two of the reference drugs. In the amastigote stage, these compounds had IC_50_ values of 12.3 μM and 50.26 μM, respectively. In contrast, Ace and Tol demonstrated greater activity against the NINOA strain, especially against the epimastigote (6.5 μM and 8.5 μM, respectively) and trypomastigote stages (46.5 μM and 9.8 μM, respectively), with the latter values outperforming all three reference drugs. Notably, Ace had promising IC_50_ values across all three developmental stages in the NINOA strain, highlighting its potential as a strong drug candidate.

Interestingly, Ace and Tol exhibited the highest selectivity index (SI), particularly against all three developmental stages in the NINOA strain (Table 3).

### 2.4. α-TcCA Enzymatic Assay

In the enzyme evaluation, Tol and Glic had no inhibitory effects against any of the three isoforms (>100 μM). Glim and Ace had the best inhibitory effects against TcCA with K_I_ values of 35.7 and 5.6 μM, respectively. However, Glim also had effects against human isoform hCAII and Ace against hCAI and hCAII (Table 4).

## 3. Discussion

CA inhibitors have been classified into classical and non-classical inhibitors based on their mechanism of action on the active site of the enzyme. Metal-chelating anions and sulfonamide derivatives represent the classical inhibitors and have been the most studied (since 1940). They have clinical applications as anti-glaucoma, diuretic, anti-epileptic, and anti-diabetic agents. Non-classical inhibitors are represented by thiocarbamates, phenols, coumarins, and polyamines among others, and are characterized by the fact that they do not bind zinc directly.

The typical chemical structure of a classical sulfonamide-derived inhibitor consists of a zinc-binding group (ZBG), a linker region (heterocyclic or benzene ring), and a variable tail region, forming tetrahedral adducts and interacting directly with zinc via the ZBG moiety [32]. However, secondary and tertiary sulfonamides are also present in several drugs in clinical use [33] and are being investigated as potential inhibitors of CAs due to their ability to act on the zinc ion, even though the sulfonamide group is not free [34]. In this sense, derived from the screening carried out in this research, the drugs belonging to the secondary sulfonamides were selected for further analysis; the other drugs classified as NSAIDs, HDACs, kinase inhibitors, and diuretics with potential binding affinity remain to be analyzed in future research. Lastly, antibacterial, antihypertensive, and antidepressant drugs have potential adverse or collateral effects on human health and, therefore, could be discarded as options for Chagas disease treatment.

Tol, Glic, Glim, and Ace are classified as first- and second-generation and are currently used in the treatment of diabetes mellitus as hypoglycaemic agents that act by stimulating pancreatic β-cells [35,36]. Molecular docking of these compounds on the active site of the enzyme (α-TcCA) yielded binding energies higher than or equal to the control compound Aaz (−6.9 kcal/mol). The sulfonamides more commonly used as inhibitors of carbonic anhydrases are methazolamide, ethoxzolamide, and Aaz, which bind the Zn ion in tetrahedral geometry in a deprotonated state, forming a network of HB including E106 and T199 (E164 and T256, equivalent in the sequence of *T. cruzi*) and T200 (T257, *T. cruzi*) [37]. Tol and Ace highlight interaction with the cofactor (Zn) in addition to T256, which orients the substrate to catalysis. Glic interacted with T256 via hydrogen bonds, as did Glim, which also interacted with H158 via π-cation bonds. These drugs also had a pattern of interactions with specific amino acid residues V179, V193, E117, L255, and T257, reported by Guzel-Akdemir et al. in 2013 [38]. These authors analyzed a series of aromatic and heterocyclic sulfonamides on a three-dimensional protein model, and marked that interaction with V155 (also found in this work) is favourable because this residue is not present in human isoforms I and II.

Glim and Glic had high trypanocidal activity against the A1 strain, particularly in the epimastigote (IC_50_ = 10.7 and 37.6 μM) and amastigote (IC_50_ = 12.3 and 50.25 μM, respectively) stages. In contrast, Ace and Tol demonstrated higher activity against the NINOA strain, especially against the epimastigote stage (IC_50_ = 6.5 and 8.5 μM), while in the trypomastigote stage, their IC_50_ values were 46.5 and 9.8 μM, respectively. It is noteworthy that all four drug candidates share a (4-methylphenyl)sulfonylurea scaffold (highlighted in the orange box, Figure 1). Apparently, a methyl or carbonyl group at the *para* position on the phenyl ring and a small aliphatic substitution on the urea moiety improve activity. However, more compounds are necessary to establish a clear structure-activity relationship. Notably, Ace was the only compound that had consistent and promising activity across all three life cycle stages in the NINOA strain, including an IC_50_ of 46 μM against the amastigote stage. Interestingly, the highest selectivity indices (SI) were also observed for Tol and Ace against the NINOA strain, surpassing the SI values of at least one or two of the reference drugs.

The selectivity of the compounds towards one strain over another is due to the heterogeneity of biological characteristics, i.e., each strain exhibits different properties in terms of infectivity, metabolic activity, enzyme expression, and drug susceptibility levels in in vitro tests, as well as the natural resistance of each strain. In addition, it has been hypothesized that the genetic diversity of the parasite may influence aspects such as disease progression, clinical presentation, and treatment outcome [39].

To confirm the mode of action of the four FDA-approved drugs, enzymatic evaluation was carried out on α-TcCA. Glim and Ace had the best values of inhibition (35.7 and 5.6 μM, respectively). Interestingly, these inhibitory effects of Ace may suggest a correlation with trypanocidal activity, mainly in the three stages of the NINOA strain. Nevertheless, Ace had inhibitory effects against human carbonic anhydrases I and II, which could lead to adverse effects such as anorexia, arrhythmias, and hypokalaemia, further exacerbating the cardiac complications commonly associated with Chagas disease. Therefore, future studies should consider strategies to modify chemical scaffolds to enhance selectivity and minimize potential off-target effects.

On the other hand, Tol, one of the most promising compounds based on its IC_50_ values, particularly against the NINOA strain, did not exhibit inhibitory activity towards the TcCA enzyme. This suggests that its anti-*T. cruzi* activity may involve a different mechanism of action, which warrants further investigation in future studies.

To our knowledge, Glic, Glim, Tol, and Ace have not been evaluated in silico, in vitro, ex vivo or enzymatically against carbonic anhydrase in any organism. However, Juarez-Saldivar et al. propose the repositioning of hypoglycaemic drugs as inhibitors of *T. cruzi* dihydrofolate reductase-thymidylate synthase (TcDHFR-TS), a protein that catalyzes the reduction of folate to tetrahydrofolate and the subsequent synthesis of thymidylate, an essential precursor of DNA synthesis [40]. Their results in an in vitro epimastigote model showed that glipizide and glyburide had the best IC_50_ values of 13.4 ± 6 and 12 ± 5 μM, respectively, similar to Bzn at 12 ± 2 μM, while glyburide had an IC_50_ of 66 ± 12 μM. Interestingly, the N-(cyclohexylcarbamoyl)benzenesulfonamide scaffold present in glipizide and glyburide was also found in Glic and Ace, raising the possibility that these compounds may have a dual mechanism of action, which would need to be confirmed by enzymatic assays. Finally, the background information found and the scheme of this work (in silico, in vitro, ex vivo and enzymatic analysis) towards hypoglycaemic drugs on α-TcCA indicate that it is feasible that FDA families not yet explored could be considered for future evaluation and repositioning projects.

## 4. Materials and Methods

### 4.1. Virtual Screening

#### Selection of FDA Compounds for Molecular Docking

The drug chemo-library was obtained from the DrugBank database, analyzed, and filtered using the DataWarrior program [41]. Duplicates were removed, and only sulfonamide derivatives were selected to obtain a curated list of drugs for subsequent molecular docking to the active site of α-TcCA. The compounds were filtered and those in the experimental or research phase were removed, resulting in a total number of drug candidates in sdf format.

### 4.2. Ligand and α-TcCA Receptor Preparation

In a Colab executable document (https://colab.research.google.com/?hl=es) [42], the following software packages and libraries were installed: MiniConda (https://docs.conda.io/projects/miniconda/en/latest/, accessed on 20 January 2023), Open Babel (3.1.1) [43], PyMol (2.5.4) (https://pymol.org/2/ accessed on 20 January 2023) [44], gnina (1.0) (https://github.com/gnina/gnina) [45], and Plip 2.3.0 or https://plip-tool.biotec.tu-dresden.de/plip-web/plip/index accessed on 18 March 2023 [46], as well as the pandas library for receptor preparation, ligand handling, molecular docking, analysis, and visualization of interactions.

The protein structure of α-TcCA was obtained from the AlphaFold database [47] (ID: Q4CVY4), while the reference carbonic anhydrase (human isoform II) or non-target was obtained from the Protein Data Bank with ID: 3B4F [48]. The preparation of the receptors was performed in PyMol (2.5.4) [49]. Hydrogen bond acceptors and donors were added, and water molecules were removed before saving the receptor files in .pdb format. Additionally, the center of mass (X, Y, and Z coordinates) was calculated in PyMol, considering the residues on the catalytic site (H158, H160, H177, E164, and T256 according 3B4F nomenclature). This information was then used to design a 20 × 20 × 20 Å box.

### 4.3. Molecular Docking Analysis

Molecular docking of the compounds obtained from DrugBank on the active site of the α-TcCA was performed using gnina 1.0 software [49]. This procedure was run considering the cofactor (Zn), residues of the catalytic triad (H158, H160 and H177), and two residues responsible for substrate orientation during catalysis (E164 and T256). To locate the residues of interest on the active site, an alignment of the other members of the α protein family was conducted, including human CA II obtained from the Protein Data Bank (PDB: 3B4F) that contain a crystallized ligand (ID: TUO; 2-(hydrazinocarbonyl)-3-phenyl-1H-indole-5-sulfonamide) on the active site. Docking validation was performed by extracting the crystallized ligand from the binding site and generating the 3D structure of the control ligand from scratch (re-docking). Subsequently, the control ligand was subjected to molecular docking with gnina 1.0, considering the best pose based on the binding energy and RMSD value (≤2 Å) [50]. For the selection of the best ligands, the binding free energy of the control drug acetazolamide (Aaz), an inhibitor agent (K_I_ = 61.6 nM) [51], was taken as the cut-off point, as well as for its interaction with residues of interest.

### 4.4. Interaction Analysis

Considering only the poses that comply with the cut-off value for binding free energy, these were complexed with their respective protein and saved in .pdb format to be subsequently analyzed to determine their non-covalent interactions with the Protein–Ligand Interaction Profile (PLIP) version 2.3.0 or in https://plip-tool.biotec.tu-dresden.de/plip-web/plip/index (accessed on 18 March 2023) [52].

Acceptance criteria for candidate ligands were based on three main characteristics: binding free energy (kcal/mol) equal to or higher than the control drug, interactions with at least one residue of interest in the active site or with the cofactor, and commercial availability (not experimental or investigational). Candidate ligands were evaluated in an in vitro model of *T. cruzi* epimastigotes from two Mexican strains, NINOA and A1, and with cytotoxicity assays in mouse macrophage cells (J774.2).

### 4.5. Trypanocidal Activity

The selected drugs were purchased from Sigma-Aldrich without further purification. For the in vitro evaluation, the methodology proposed by Domínguez-Diaz et al. 2021 [53] against epimastigotes of *T. cruzi* strains NINOA (MHOM/MX/1994/NINOA) and A1 for the evaluation of candidate compounds was used. Both strains were maintained in liver-infusion tryptose (LIT) medium supplemented with 10% fetal bovine serum (FBS) and 0.1% penicillin–streptomycin. These strains were maintained by transferring 1 × 10^6^ parasites/mL to fresh medium once a week. Candidate compounds and controls Nfx, Bzn, and Aaz were evaluated. All compounds were initially prepared at a concentration of 10 mg/mL using dimethyl sulfoxide (DMSO) as a diluent. Serial dilutions were then performed with LIT medium until concentrations of 100 to 0.46 μg/mL of each compound were obtained. Amounts of 1 × 10^6^ *T. cruzi* epimastigotes were grown in each well and incubated for 48 h at 28 °C in a final volume of 200 μL. DMSO was included as a negative control, and reference drugs as positive controls. At the end of the incubation period, 20 μL of resazurin solution (2.5 mM) was added to each well and incubated for 3 h. All assays were performed in triplicate, and the IC_50_ value was determined by probit analysis [53].

### 4.6. Ex Vivo Analysis

Ex vivo evaluation of the trypanocidal activity of the tested compounds was conducted according to previously established protocols [53,54]. Blood was collected via intracardiac puncture from mice infected with *T. cruzi* NINOA and A1 strains and diluted in phosphate-buffered saline (PBS, pH 7.2) to achieve a final concentration of 2 × 10^6^ blood trypomastigotes/mL. A volume of 195 μL of this suspension was added to each well of a 96-well plate, followed by the addition of 5 μL of the compounds at varying concentrations (ranging from 200 μM to 3.125 μM). The plates were incubated at 4 °C for 24 h. A 0.2% DMSO solution was used as negative control, while Nfx and Bzn served as positive controls. Following incubation, live parasites were quantified using a Neubauer chamber, and the half-maximal inhibitory concentration (IC_50_) was determined by probit analysis. All assays were performed in three independent experiments, each conducted in triplicate.

### 4.7. Amastigote Assay

Amastigote susceptibility to reference drugs was evaluated in vitro. In brief, 5 × 10^4^ murine macrophages (cell line J774.2, ATCC ^®^TIB-67) and stationary epimastigotes from parasite isolates, at a 1:10 ratio, were seeded in 200 µL/well of RPMI-1640 culture medium supplemented with 10% heat-inactivated FBS and 100 U/mL penicillin plus 100 µg/mL streptomycin in 96-well plates and incubated for 48 h at 37 °C and 5% CO_2_ in a humidity chamber. The cells were then washed several times with RPMI medium to remove free non-infective epimastigotes, and the final washing medium was replaced with 200 µL/well of culture medium containing different concentrations of Bzn and Nfx (100 μM to 3.125 μM). After plates were incubated for another 48 h at 37 °C in 5% CO_2_, the culture medium was replaced with an equal volume of lysis solution (RPMI-1640 and 0.01% SDS) and maintained at room temperature for 20 min. The lysis solution was then replaced with Schneider’s medium followed by incubation at 28 °C for another 4 to 5 days to allow the transformation of viable amastigotes and their subsequent proliferation. Then, MTT colorimetric assays were performed. Finally, the half maximal inhibitory concentration (IC_50_) was determined by probit analysis. All studies were approved by the Institutional Committee for Handling and Animal Care of the Facultad de Ciencias Quimicas de la Universidad Autonoma “Benito Juarez” de Oaxaca (Register FCQ-CIP-0209243, Approved 23 September 2024) and were carried out according to Mexican guidance (NOM-062-ZOO-1999).

### 4.8. Cytotoxicity in Murine Macrophages

Cytotoxicity assays were performed using the methodology proposed by Domínguez-Diaz et al. [53] on a mouse macrophage cell line (J774.2). Cells were cultured in LIT medium supplemented with 10% SFB, penicillin (100 U μg/mL), and streptomycin (100 U μg/mL) at 37 °C in a 5% CO_2_ atmosphere. The culture medium was replaced at 2–3 day intervals. To evaluate the cytotoxicity of the compounds, 50,000 cells/well were plated in a 96-well plate and allowed to adhere for 24 h at 37 °C. Subsequently, the compounds were added at concentrations of 0.8 to 100 μg/mL to obtain a final volume of 200 μL and incubated for 48 to 37 h with 5% CO_2_. DMSO 0.1% (maximum concentration used) was included as a negative control and the control drugs as positive ones. The metabolic activity of the cell was determined following the MTT method, the % cell viability was calculated, and the median lethal concentration (LC_50_) was determined by Probit analysis [53]. Analyses were performed in triplicate, and the Selectivity Index (SI) for the three parasitic stages in the NINOA and A1 strains (CC_50_/IC_50_) was calculated.

### 4.9. Carbonic Anhydrase Enzymatic Assays

An Applied Photophysics stopped-flow instrument was used for assaying the CA-catalysed CO_2_ hydration activity [31]. Phenol red (at a concentration of 0.2 mM) was used as an indicator, working at the absorbance maximum of 557 nm, with 20 mM Hepes (pH 7.5) as a buffer and 20 mM Na_2_SO_4_ (for maintaining constant the ionic strength), following the initial rates of the CA-catalysed CO_2_ hydration reaction for a period of 10–100 s. The CO_2_ concentrations ranged from 1.7 to 17 mM for the determination of the kinetic parameters and inhibition constants. For each inhibitor, at least six traces of the initial 5–10% of the reaction were used for determining the initial velocity. The uncatalyzed rates were determined in the same manner and subtracted from the total observed rates. Stock solutions of inhibitor (0.1 mM) were prepared in distilled/deionized water, and dilutions up to 0.01 nM were performed thereafter with the assay buffer. Inhibitor and enzyme solutions were preincubated together for 15 min at room temperature before assaying to allow for the formation of the E–I complex. The inhibition constants were obtained by nonlinear least-squares methods using PRISM 3 and the Cheng–Prusoff equation and represent the mean from at least three different determinations. The enzyme concentrations were in the range of 6–14 nM. All hCA isoforms were recombinant ones obtained in-house, as reported earlier [55].

## 5. Conclusions

This study highlights the potential of FDA-approved sulfonamide-based drugs, originally developed as hypoglycaemic agents, for repurposing as trypanocidal compounds targeting α-TcCA. Through in silico,– in vitro, ex vivo, and enzymatic evaluations, four candidates, Tol, Glic, Glim, and Ace were identified with promising activity against distinct life stages and strains of *T. cruzi*. Notably, Ace demonstrated consistent trypanocidal efficacy across all three stages in the NINOA strain and had enzymatic inhibition of α-TcCA as the mode of action, although it’s known off-target effects on human CA II warrant further structural optimization. Conversely, Tol activity appears to be mediated through a different molecular target, underscoring the potential for alternative mechanisms beyond CA inhibition. To our knowledge, this work provides new insights into the repositioning of sulfonamide-based drugs and underscores the relevance of integrating docking studies, enzymatic assays, and parasitological evaluations to identify and optimize selective and effective agents against Chagas disease. Future research should focus on improving target selectivity, reducing host toxicity, and further elucidating alternative mechanisms of action to advance these compounds toward clinical application.

## Figures and Tables

**Table 1:** FDA-approved drugs with BFE values above the cut-off point (–6.9 kcal/mol) set by acetazolamide (**Aaz**).

	DB ID	BFE (kcal/mol)			DB ID	BFE (kcal/mol)	
1	DB00222	−8.9		24	DB00580	−7.4	
2	DB11395	−8.8		25	DB13773	−7.3	
3	DB00214	−8.4		26	DB00436	−7.3	
4	DB05015	−8.1		27	DB15861	−7.3	
5	DB00774	−8		28	DB01120	−7.3	
6	DB06268	−8		29	DB11739	−7.3	
7	DB00310	−7.9		30	DB01015	−7.2	
8	DB08881	−7.8		31	DB00554	−7.2	
9	DB00414	−7.8		32	DB11462	−7.2	
10	DB14033	−7.7		33	DB11464	−7.2	
11	DB08942	−7.7		34	DB00880	−7.1	
12	DB00278	−7.7		35	DB06150	−7.1	
13	DB00482	−7.7		36	DB01298	−7.1	
14	DB11461	−7.6		37	DB08439	−7.1	
15	DB00263	−7.5		38	DB01325	−7.1	
16	DB00999	−7.4		39	DB00814	−7.1	
17	DB00695	−7.4		40	DB08912	−7	
18	DB13532	−7.4		41	DB00606	−7	
19	DB06729	−7.4		42	DB01021	−7	
20	DB09289	−7.4		43	DB13165	−7	
21	DB14973	−7.4		44	DB09215	−6.9	
22	DB13284	−7.4		45	DB01124	−6.9	
23	DB00808	−7.4		46	DB00819_Aaz	−6.9	
		Hypoglycaemics				NSAIDs	
		Diuretics				Antihypertensives	
		Antibacterials				Antidepressants	
		Vet approved				Histone deacetylase (HDAC), kinase and COX-2 inhibitor	

**Table 2:** Trypanocidal activity of four sulfonamide-based FDA-approved drugs against the three parasite stages in the NINOA and A1 strains, and cytotoxic activity against macrophages.

ID	Epimastigote (In Vitro)		Trypomastigote (Ex Vivo)		Amastigote (Ex Vivo)		J774.2 ^b^ CC_50_ (μM ± SD)
	NINOA	A1	NINOA	A1	NINOA	A1	
		^a^ IC_50_ (μM ± SD)					
Glic	>200	10.7 ± 1.5	89.4 ± 0.1	>200	>200	12.3 ± 0.01	>200
Glim	70.2 ± 3.2	37.6 ± 1.5	>200	>200	>200	50.26 ± 0.01	14.2 ± 2.3
Ace	6.5 ± 2.1	52.9 ± 2.7	46.5 ± 0.1	>200	46 ± 0.6	>200	>200
Tol	8.5 ± 1.4	>200	9.8 ± 0.1	97.7 ± 0.1	148.7 ± 0.01	44.5 ± 0.01	>200
Reference drugs							
Aaz	5.5 ± 1.7	15.2 ± 2.8	198 ± 0.1	>200	>200	9.6 ± 0.01	12.6 ± 4.7
Nfx	7.1 ± 0.1	39.1 ± 0.07	156 ± 0.1	118.2 ± 0.02	70.5 ± 0.6	54.8 ± 0.01	164.2 ± 0.3
Bzn	30.3 ± 0.03	19.3 ± 0.08	167.1 ± 0.03	145.3 ± 0.2	Ne	Ne	133.9 ± 0.06
		Did not exceed drug controls				Exceeded one or two drug controls	

^a^ IC_50_: Half-maximal inhibitory concentration. ^b^ CC_50_: Half-maximal cytotoxicity concentration. Ne: Not evaluated.

**Table 3:** Selectivity index (SI) of four sulfonamide-based FDA-approved drugs towards parasitic stages.

^a^ SI						
ID	Epimastigote		Trypomastigote		Amastigote	
	NINOA	A1	NINOA	A1	NINOA	A1
Glic	^b^ Nc	>18.7	>2.2	^b^ Nc	^b^ Nc	>16.3
Glim	0.2	0.38	0.07	0.07	0.07	0.3
Ace	>30.7	>3.8	>4.3	^b^ Nc	>4.34	^b^ Nc
Tol	>23.5	^b^ Nc	>20.4	>2.0	>1.3	>4.5
Reference drugs						
Aaz	2.3	0.8	0.06	0.06	0.06	1.3
Nfx	23.2	4.2	1.0	1.4	2.3	3
Bzn	4.4	6.9	0.8	0.9	^c^ Ne	^c^ Ne

^a^ SI: selective index (CC50/IC50). ^b^ Nc: Not calculated (because IC_50_ was >200). ^c^ Ne: Not evaluated.

**Table 4:** Inhibition of human isoforms hCA I, II, and protozoan isoform α-TcCA by a CO_2_ hydrase stopped-flow assay using Aaz as a reference drug [31].

Compound	K_I_ (μM) ^a^		
	hCA I	hCA II	TcCA
Glic	>100	>100	>100
Glim	>100	19.9	35.7
Ace	17.3	2.9	5.6
Tol	>100	>100	>100
Aaz	0.25	0.012	0.061

^a^ Mean from three different assays by stopped-flow technique (errors were in the range of ±5–10% of the reported values).

## Data Availability

Data availability statements are available in the Supplementary Materials.

## Supplementary Materials

The following supporting information can be downloaded at: https://www.mdpi.com/article/10.3390/ph18050669/s1, Table S1: Sulfonamide-derived drugs filtered in DataWarrior; Table S2: Commercially available compounds; Figure S1: Alignment of proteins belonging to the α family; Figure S2: Interaction profile of Aaz; Figure S3: Interaction profile of 45 candidate compounds at the active site of α-TcCA.

## Abbreviations

The following abbreviations are used in this manuscript.

α-TcCA	α-*Trypanosoma cruzi* Carbonic Anhydrase
Aaz	Acetazolamide
Ace	Acetohexamide
CC_50_	Half-maximal cytotoxicity concentration
DB	Drug bank
BFE	Binding free energy
FDA	Food and Drug Administration
Glic	Gliclazide
Glim	Glimepiride
HB	Hydrogen bond
HI	Hydrophobic interaction
IC_50_	Half-maximal inhibitory concentration
K_I_	Inhibitory constant
NSAID	Nonsteroidal anti-inflammatory drug
PDB	Protein data bank
π-c	Pi-cation
PLIP	Protein–Ligand Interaction Profile
SB	Salt bridge
SI	Selectivity index
Tol	Tolbutamide
Zn	Zinc
