# Supplementary material for: Repositioning FDA-Approved Sulfonamide-Based Drugs as Potential Carbonic Anhydrase Inhibitors in Trypanosoma cruzi: Virtual Screening and In Vitro Studies
Source: Pharmaceuticals (Basel). 2025 May 1;18(5):669. doi: 10.3390/ph18050669 (PMC12114598; doi:10.3390/ph18050669)

Tittle

Repositioning FDA Drugs as Potential Carbonic Anhydrase Inhibitors from *Trypanosoma cruzi*: Virtual Screening and *In Vitro* Studies

Eyra Ortiz-Pérez[1], Adriana Moreno Rodríguez[2], Timoteo Delgado-Maldonado[1], Jessica L. Ortega-Balleza[1], Alonzo González-González[1], Alma. D. Paz-Gonzalez[1], Karina Vázquez[3], Guadalupe Avalos-Navarro, Simone Giovannuzzi[5], Claudiu T. Supuran[5], Gildardo Rivera[1*]

Supplementary material

Table S1. Sulphonamide-derived drugs filtered in DataWarrior and evaluated by molecular docking on the active site of α-*Tc*CA. Candidate drugs are highlighted in grey.

	DB ID	BFE (Kcal/mol)
**1**	DB00222_Glim	-8.9
**2**	DB11395	-8.8
**3**	DB07861	-8.4
**4**	DB00214	-8.4
**5**	DB15287	-8.3
**6**	DB04238	-8.2
**7**	DB03046	-8.2
**8**	DB05015	-8.1
**9**	DB07363	-8.0
**10**	DB15229	-8.0
**11**	DB06921	-8.0
**12**	DB07325	-8.0
**13**	DB00774	-8.0
**14**	DB07261	-8.0
**15**	DB07697	-8.0
**16**	DB06268	-8.0
**17**	DB02071	-7.9
**18**	DB07211	-7.9
**19**	DB00310	-7.9
**20**	DB14801	-7.9
**21**	DB08046	-7.9
**22**	DB07024	-7.8
**23**	DB08881	-7.8
**24**	DB07290	-7.8
**25**	DB07321	-7.8
**26**	DB01877	-7.8
**27**	DB07685	-7.8
**28**	DB04698	-7.8
**29**	DB00414_Aceto	-7.8
**30**	DB06629	-7.7
**31**	DB14033	-7.7
**32**	DB12378	-7.7
**33**	DB16066	-7.7
**34**	DB16103	-7.7
**35**	DB08248	-7.7
**36**	DB07312	-7.7
**37**	DB02827	-7.7
**38**	DB08942	-7.7
**39**	DB07270	-7.7
**40**	DB02033	-7.7
**41**	DB00278	-7.7
**42**	DB06999	-7.7
**43**	DB07686	-7.7
**44**	DB04140	-7.7
**45**	DB00482	-7.7
**46**	DB07000	-7.6
**47**	DB12375	-7.6
**48**	DB07136	-7.6
**49**	DB11844	-7.6
**50**	DB08162	-7.6
**51**	DB07273	-7.6
**52**	DB16078	-7.6

**53**	DB11461	-7.6
**54**	DB13051	-7.6
**55**	DB07556	-7.6
**56**	DB12282	-7.6
**57**	DB07369	-7.6
**58**	DB07999	-7.6
**59**	DB12068	-7.6
**60**	DB07568	-7.5
**61**	DB08560	-7.5
**62**	DB12649	-7.5
**63**	DB07964	-7.5
**64**	DB16114	-7.5
**65**	DB07534	-7.5
**66**	DB07527	-7.5
**67**	DB03202	-7.5
**68**	DB14059	-7.5
**69**	DB07605	-7.5
**70**	DB08211	-7.5
**71**	DB07414	-7.5
**72**	DB07934	-7.5
**73**	DB07522	-7.5
**74**	DB00263	-7.5
**75**	DB07540	-7.5
**76**	DB08631	-7.5
**77**	DB08000	-7.5
**78**	DB06891	-7.5
**79**	DB02479	-7.5
**80**	DB07583	-7.5
**81**	DB07748	-7.5
**82**	DB04588	-7.5
**83**	DB12399	-7.5
**84**	DB00999	-7.4
**85**	DB01967	-7.4
**86**	DB00695	-7.4
**87**	DB08170	-7.4
**88**	DB13532	-7.4
**89**	DB12476	-7.4
**90**	DB07687	-7.4
**91**	DB12921	-7.4
**92**	DB06729	-7.4
**93**	DB09289	-7.4
**94**	DB14973	-7.4
**95**	DB08441	-7.4

**96**	DB04244	-7.4
**97**	DB02752	-7.4
**98**	DB13284	-7.4
**99**	DB07220	-7.4
**100**	DB06370	-7.4
**101**	DB00808	-7.4
**102**	DB00580	-7.4
**103**	DB07670	-7.4
**104**	DB06835	-7.4
**105**	DB15319	-7.4
**106**	DB07572	-7.4
**107**	DB08265	-7.4
**108**	DB00839	-7.4
**109**	DB15106	-7.4
**110**	DB08527	-7.3
**111**	DB03950	-7.3
**112**	DB07724	-7.3
**113**	DB07800	-7.3
**114**	DB08487	-7.3
**115**	DB13773	-7.3
**116**	DB06140	-7.3
**117**	DB02350	-7.3
**118**	DB13675	-7.3
**119**	DB04707	-7.3
**120**	DB00436	-7.3
**121**	DB15861	-7.3
**122**	DB07927	-7.3
**123**	DB08094	-7.3
**124**	DB03526	-7.3
**125**	DB13485	-7.3
**126**	DB07732	-7.3
**127**	DB01745	-7.3
**128**	DB01120_Glic	-7.3
**129**	DB07323	-7.3
**130**	DB11739	-7.3
**131**	DB07531	-7.3
**132**	DB03656	-7.2
**133**	DB04125	-7.2
**134**	DB07252	-7.2
**135**	DB15325	-7.2
**136**	DB02917	-7.2
**137**	DB01015	-7.2
**138**	DB08962	-7.2

**139**	DB07730	-7.2
**140**	DB07126	-7.2
**141**	DB12574	-7.2
**142**	DB00554	-7.2
**143**	DB07770	-7.2
**144**	DB07688	-7.2
**145**	DB02744	-7.2
**146**	DB15316	-7.2
**147**	DB12382	-7.2
**148**	DB07947	-7.2
**149**	DB11772	-7.2
**150**	DB07746	-7.2
**151**	DB07520	-7.2
**152**	DB11462	-7.2
**153**	DB11464	-7.2
**154**	DB07322	-7.2
**155**	DB13430	-7.2
**156**	DB12254	-7.1
**157**	DB02858	-7.1
**158**	DB00880	-7.1
**159**	DB07692	-7.1
**160**	DB05562	-7.1
**161**	DB08645	-7.1
**162**	DB06150	-7.1
**163**	DB07309	-7.1
**164**	DB07056	-7.1
**165**	DB03818	-7.1
**166**	DB16962	-7.1
**167**	DB11992	-7.1
**168**	DB01298	-7.1
**169**	DB15097	-7.1
**170**	DB07995	-7.1
**171**	DB07876	-7.1
**172**	DB03693	-7.1
**173**	DB15024	-7.1
**174**	DB08439	-7.1
**175**	DB07541	-7.1
**176**	DB01325	-7.1
**177**	DB12863	-7.1
**178**	DB07848	-7.1
**179**	DB08609	-7.1
**180**	DB07944	-7.1
**181**	DB00814	-7.1
**182**	DB16044	-7.1
**183**	DB12022	-7.1
**184**	DB04012	-7.1
**185**	DB15630	-7.1
**186**	DB08912	-7.0
**187**	DB00606	-7.0
**188**	DB01021	-7.0
**189**	DB08481	-7.0
**190**	DB15250	-7.0
**191**	DB07115	-7.0
**192**	DB05943	-7.0
**193**	DB08426	-7.0
**194**	DB08213	-7.0
**195**	DB13165	-7.0
**196**	DB08495	-7.0
**197**	DB13988	-7.0
**198**	DB08132	-7.0
**199**	DB07798	-7.0
**200**	DB13803	-7.0
**201**	DB08961	-7.0
**202**	DB09215	-6.9
**203**	DB01124_Tol	-6.9
**204**	DB00819_Aaz	-6.9
	**Cut-off**	
**205**	DB16227	-6.9
**206**	DB16212	-6.9
**207**	DB02118	-6.9
**208**	DB07050	-6.9
**209**	DB07739	-6.9
**210**	DB08271	-6.9
**211**	DB00576	-6.9
**212**	DB07233	-6.9
**213**	DB11389	-6.9
**214**	DB07710	-6.9
**215**	DB13020	-6.9
**216**	DB11893	-6.9
**217**	DB12505	-6.9
**218**	DB04763	-6.9
**219**	DB04232	-6.9
**220**	DB15047	-6.9
**221**	DB01712	-6.9
**222**	DB08278	-6.9
**223**	DB07817	-6.9

**224**	DB07169	-6.9
**225**	DB07996	-6.9
**226**	DB00562	-6.9
**227**	DB11441	-6.9
**228**	DB08301	-6.8
**229**	DB07254	-6.8
**230**	DB07533	-6.8
**231**	DB03262	-6.8
**232**	DB06321	-6.8
**233**	DB08603	-6.8
**234**	DB12623	-6.8
**235**	DB13405	-6.8
**236**	DB00775	-6.8
**237**	DB03207	-6.8
**238**	DB07313	-6.8
**239**	DB08223	-6.8
**240**	DB14856	-6.8
**241**	DB02367	-6.8
**242**	DB07847	-6.8
**243**	DB02866	-6.8
**244**	DB00869	-6.8
**245**	DB01737	-6.8
**246**	DB07986	-6.8
**247**	DB12135	-6.8
**248**	DB07632	-6.8
**249**	DB00549	-6.8
**250**	DB05095	-6.8
**251**	DB06506	-6.8
**252**	DB04032	-6.8
**253**	DB13283	-6.7
**254**	DB07747	-6.7
**255**	DB07920	-6.7
**256**	DB13708	-6.7
**257**	DB06677	-6.7
**258**	DB08329	-6.7
**259**	DB08640	-6.7
**260**	DB11961	-6.7
**261**	DB06309	-6.7
**262**	DB06951	-6.7
**263**	DB07275	-6.7
**264**	DB01623	-6.7
**265**	DB12196	-6.7
**266**	DB04676	-6.7

**267**	DB13663	-6.7
**268**	DB13036	-6.7
**269**	DB01119	-6.7
**270**	DB07080	-6.7
**271**	DB03596	-6.7
**272**	DB02610	-6.7
**273**	DB07121	-6.7
**274**	DB16877	-6.7
**275**	DB03598	-6.7
**276**	DB08003	-6.7
**277**	DB09355	-6.7
**278**	DB00524	-6.7
**279**	DB13320	-6.7
**280**	DB07664	-6.7
**281**	DB06725	-6.7
**282**	DB07691	-6.7
**283**	DB07333	-6.7
**284**	DB12419	-6.7
**285**	DB06943	-6.7
**286**	DB16304	-6.6
**287**	DB02986	-6.6
**288**	DB07538	-6.6
**289**	DB01582	-6.6
**290**	DB04549	-6.6
**291**	DB13214	-6.6
**292**	DB07683	-6.6
**293**	DB13989	-6.6
**294**	DB07843	-6.6
**295**	DB06844	-6.6
**296**	DB03307	-6.6
**297**	DB05038	-6.6
**298**	DB00489	-6.6
**299**	DB06821	-6.6
**300**	DB01264	-6.6
**301**	DB08659	-6.6
**302**	DB02861	-6.6
**303**	DB05549	-6.6
**304**	DB02220	-6.6
**305**	DB08573	-6.6
**306**	DB15883	-6.6
**307**	DB12233	-6.6
**308**	DB07277	-6.5
**309**	DB00909	-6.5

**310**	DB08798	-6.5
**311**	DB01144	-6.5
**312**	DB00706	-6.5
**313**	DB11718	-6.5
**314**	DB16319	-6.5
**315**	DB07175	-6.5
**316**	DB03558	-6.5
**317**	DB15326	-6.5
**318**	DB12263	-6.5
**319**	DB00276	-6.5
**320**	DB02973	-6.5
**321**	DB05407	-6.5
**322**	DB03477	-6.5
**323**	DB04081	-6.5
**324**	DB12614	-6.5
**325**	DB05884	-6.5
**326**	DB11362	-6.5
**327**	DB17077	-6.5
**328**	DB13406	-6.5
**329**	DB07922	-6.5
**330**	DB01581	-6.5
**331**	DB07505	-6.5
**332**	DB11817	-6.5
**333**	DB06836	-6.5
**334**	DB08605	-6.5
**335**	DB11736	-6.5
**336**	DB03221	-6.5
**337**	DB08157	-6.5
**338**	DB00634	-6.5
**339**	DB08126	-6.4
**340**	DB07997	-6.4
**341**	DB14839	-6.4
**342**	DB04503	-6.4
**343**	DB00952	-6.4
**344**	DB01098	-6.4
**345**	DB01289	-6.4
**346**	DB00701	-6.4
**347**	DB03333	-6.4
**348**	DB02069	-6.4
**349**	DB00887	-6.4
**350**	DB02197	-6.4
**351**	DB00308	-6.4
**352**	DB12708	-6.4
**353**	DB08599	-6.4
**354**	DB00891	-6.4
**355**	DB11719	-6.4
**356**	DB08556	-6.4
**357**	DB08122	-6.4
**358**	DB03468	-6.4
**359**	DB07742	-6.4
**360**	DB08155	-6.4
**361**	DB06589	-6.4
**362**	DB11547	-6.4
**363**	DB01622	-6.4
**364**	DB00918	-6.4
**365**	DB07278	-6.4
**366**	DB07062	-6.3
**367**	DB04089	-6.3
**368**	DB04772	-6.3
**369**	DB08156	-6.3
**370**	DB01748	-6.3
**371**	DB00664	-6.3
**372**	DB00469	-6.3
**373**	DB03294	-6.3
**374**	DB08107	-6.3
**375**	DB02602	-6.3
**376**	DB14628	-6.3
**377**	DB00391	-6.3
**378**	DB08729	-6.3
**379**	DB04371	-6.3
**380**	DB15071	-6.3
**381**	DB08134	-6.3
**382**	DB07844	-6.3
**383**	DB13135	-6.3
**384**	DB07921	-6.3
**385**	DB16623	-6.3
**386**	DB08133	-6.3
**387**	DB02221	-6.3
**388**	DB12392	-6.3
**389**	DB01299	-6.3
**390**	DB08202	-6.3
**391**	DB12557	-6.3
**392**	DB08106	-6.3
**393**	DB04180	-6.3
**394**	DB03039	-6.3
**395**	DB00948	-6.3

**396**	DB07804	-6.3
**397**	DB11761	-6.3
**398**	DB03124	-6.3
**399**	DB08451	-6.3
**400**	DB03877	-6.2
**401**	DB07547	-6.2
**402**	DB00669	-6.2
**403**	DB04513	-6.2
**404**	DB07544	-6.2
**405**	DB07791	-6.2
**406**	DB16021	-6.2
**407**	DB06862	-6.2
**408**	DB08765	-6.2
**409**	DB05245	-6.2
**410**	DB08083	-6.2
**411**	DB08644	-6.2
**412**	DB12473	-6.2
**413**	DB15975	-6.2
**414**	DB12234	-6.2
**415**	DB00204	-6.2
**416**	DB08125	-6.2
**417**	DB01888	-6.2
**418**	DB07467	-6.2
**419**	DB00359	-6.2
**420**	DB02429	-6.2
**421**	DB08165	-6.2
**422**	DB07476	-6.2
**423**	DB01382	-6.2
**424**	DB03844	-6.2
**425**	DB13726	-6.2
**426**	DB07429	-6.2
**427**	DB08029	-6.2
**428**	DB13248	-6.2
**429**	DB00232	-6.1
**430**	DB12500	-6.1
**431**	DB08484	-6.1
**432**	DB12418	-6.1
**433**	DB13580	-6.1
**434**	DB01194	-6.1
**435**	DB13792	-6.1
**436**	DB07238	-6.1
**437**	DB15882	-6.1
**438**	DB01067	-6.1

**439**	DB07162	-6.1
**440**	DB15055	-6.0
**441**	DB11941	-6.0
**442**	DB01032	-6.0
**443**	DB02723	-6.0
**444**	DB00672	-6.0
**445**	DB08123	-6.0
**446**	DB07446	-6.0
**447**	DB01319	-6.0
**448**	DB01964	-6.0
**449**	DB13617	-6.0
**450**	DB12819	-6.0
**451**	DB08782	-6.0
**452**	DB01836	-6.0
**453**	DB07772	-6.0
**454**	DB11843	-6.0
**455**	DB16040	-6.0
**456**	DB07713	-6.0
**457**	DB02535	-5.9
**458**	DB07526	-5.9
**459**	DB03270	-5.9
**460**	DB14708	-5.9
**461**	DB00311	-5.9
**462**	DB02449	-5.9
**463**	DB08105	-5.9
**464**	DB12703	-5.9
**465**	DB07048	-5.9
**466**	DB08494	-5.9
**467**	DB04416	-5.9
**468**	DB08304	-5.9
**469**	DB08967	-5.9
**470**	DB07114	-5.9
**471**	DB13523	-5.9
**472**	DB01324	-5.9
**473**	DB08108	-5.9
**474**	DB00705	-5.9
**475**	DB05490	-5.9
**476**	DB08673	-5.8
**477**	DB03697	-5.8
**478**	DB14976	-5.8
**479**	DB04593	-5.8
**480**	DB06147	-5.8
**481**	DB07508	-5.8

**482**	DB02925	-5.8
**483**	DB06933	-5.8
**484**	DB08112	-5.8
**485**	DB04767	-5.7
**486**	DB12836	-5.7
**487**	DB04203	-5.7
**488**	DB05188	-5.7
**489**	DB03890	-5.7
**490**	DB01689	-5.7
**491**	DB04002	-5.7
**492**	DB02087	-5.6
**493**	DB00559	-5.6
**494**	DB06267	-5.6
**495**	DB17050	-5.6
**496**	DB13699	-5.6
**497**	DB00862	-5.6
**498**	DB17299	-5.6
**499**	DB16761	-5.6
**500**	DB07624	-5.5
**501**	DB11392	-5.5
**502**	DB05100	-5.5
**503**	DB17171	-5.5
**504**	DB13547	-5.5
**505**	DB17117	-5.5
**506**	DB06795	-5.5
**507**	DB08341	-5.5
**508**	DB08039	-5.5
**509**	DB01621	-5.5
**510**	DB02055	-5.5
**511**	DB12889	-5.5
**512**	DB06558	-5.5
**513**	DB12285	-5.5
**514**	DB04758	-5.5
**515**	DB13556	-5.4
**516**	DB15108	-5.4
**517**	DB01784	-5.4
**518**	DB03583	-5.4
**519**	DB12367	-5.4
**520**	DB00703	-5.4
**521**	DB00259	-5.4
**522**	DB02689	-5.4
**523**	DB06970	-5.4
**524**	DB14983	-5.3

**525**	DB03081	-5.3
**526**	DB11408	-5.3
**527**	DB11692	-5.3
**528**	DB04142	-5.2
**529**	DB07790	-5.2
**530**	DB05495	-5.2
**531**	DB11871	-5.2
**532**	DB03031	-5.1
**533**	DB04607	-5.1
**534**	DB01016	-5.1
**535**	DB06974	-5.1
**536**	DB16147	-5.1
**537**	DB05817	-5.0
**538**	DB15124	-5.0
**539**	DB14929	-4.9
**540**	DB15959	-4.9
**541**	DB15187	-4.9
**542**	DB08488	-4.8
**543**	DB04855	-4.8
**544**	DB08746	-4.8
**545**	DB00203	-4.8
**546**	DB03157	-4.8
**547**	DB05961	-4.7
**548**	DB12149	-4.7
**549**	DB08305	-4.6
**550**	DB12428	-4.6
**551**	DB08745	-4.6
**552**	DB12548	-4.6
**553**	DB07216	-4.5
**554**	DB03768	-4.4
**555**	DB11663	-4.4
**556**	DB12297	-4.4
**557**	DB04606	-4.3
**558**	DB11861	-4.3
**559**	DB12800	-4.2
**560**	DB08270	-4.2
**561**	DB08303	-4.2
**562**	DB01347	-4.1
**563**	DB16162	-4.1
**564**	DB11792	-4.0
**565**	DB00932	-4.0
**566**	DB16236	-3.9
**567**	DB13929	-3.9

**568**	DB12805	-3.8
**569**	DB00795	-3.7
**570**	DB01251	-3.5
**571**	DB16262	-3.5
**572**	DB02411	-3.3
**573**	DB12717	-3.3
**574**	DB01879	-3.0
**575**	DB06367	-3.0
**576**	DB07455	-2.8
**577**	DB11902	-2.7
**578**	DB04172	-2.7
**579**	DB04427	-2.5
**580**	DB02259	-2.3
**581**	DB03141	-2.2
**582**	DB12272	-2.0
**583**	DB15177	-1.9
**584**	DB12340	-1.9
**585**	DB13052	-1.7
**586**	DB04748	-1.7
**587**	DB03311	-1.7
**588**	DB04887	-1.6
**589**	DB12051	-1.4
**590**	DB07019	-1.3
**591**	DB13000	-0.9
**592**	DB15499	-0.7
**593**	DB03642	-0.6
**594**	DB16843	-0.5
**595**	DB12702	-0.4
**596**	DB12643	-0.1
**597**	DB15646	0.1
**598**	DB15791	0.4
**599**	DB17235	0.5
**600**	DB15444	0.6
**601**	DB12724	0.7

**602**	DB06350	1.3
**603**	DB05476	2.0
**604**	DB13095	2.4
**605**	DB14923	3.3
**606**	DB16095	4.0
**607**	DB15156	4.0
**608**	DB12165	4.1
**609**	DB16261	4.1
**610**	DB15426	4.4
**611**	DB15623	5.0
**612**	DB01630	6.1
**613**	DB14946	6.1
**614**	DB11586	6.2
**615**	DB17109	8.0
**616**	DB09183	8.5
**617**	DB12069	9.4
**618**	DB13101	9.8
**619**	DB04673	10.8
**620**	DB12655	12.5
**621**	DB11779	16.0
**622**	DB06290	16.4
**623**	DB17166	26.8
**624**	DB16285	28.4
**625**	DB09297	29.4
**626**	DB12785	30.4
**627**	DB15673	32.3
**628**	DB11927	40.9
**629**	DB11929	41.5
**630**	DB12026	52.9
**631**	DB15371	57.3
**632**	DB11575	58.6
**633**	DB15249	64.7
**634**	DB13879	69.6
**635**	DB17219	84.4

Table S2. Selection of commercially available compounds for human and animal use (light blue), to discard those that are at the experimental or investigational stage.

	PDB ID	BFE (Kcal/mol)	Background
**1**	DB00222_Glim	-8.9	Glimepiride sulfonylurea drug used to treat type 2 diabetes mellitus.
**2**	DB11395	-8.8	Deracoxib non-steroidal anti-inflammatory drug of the coxib class, used in veterinary medicine to treat osteoarthritis in dogs.
**3**	DB07861	-8.4	Experimental
**4**	DB00214	-8.4	Torsemide diuretic used to treat hypertension and edema associated with heart failure, renal failure, or liver disease.
**5**	DB15287	-8.3	Investigational
**6**	DB04238	-8.2	Experimental
**7**	DB03046	-8.2	Experimental
**8**	DB05015	-8.1	Belinostat is a histone deacetylase (HDAC) inhibitor used for the treatment of patients with relapsed or refractory peripheral T-cell lymphoma (PTCL).
**9**	DB07363	-8	Experimental
**10**	DB15229	-8	Investigational
**11**	DB06921	-8	Experimental
**12**	DB07325	-8	Experimental
**13**	DB00774	-8	Hydroflumethiazide is a thiazide diuretic used to treat hypertension as well as edema due to congestive heart failure and liver cirrhosis.
**14**	DB07261	-8	Experimental
**15**	DB07697	-8	Experimental
**16**	DB06268	-8	Sitaxentan was marketed under the trade name Thelin for the treatment of pulmonary arterial hypertension (PAH)
**17**	DB02071	-7.9	Experimental
**18**	DB07211	-7.9	Experimental
**19**	DB00310	-7.9	Chlorthalidone is a diuretic used to treat hypertension or edema caused by heart failure, renal failure, hepatic cirrhosis, estrogen therapy, and other conditions.
**20**	DB14801	-7.9	Investigational
**21**	DB08046	-7.9	Experimental
**22**	DB07024	-7.8	Experimental
**23**	DB08881	-7.8	Vemurafenib is a kinase inhibitor used to treat patients with Erdheim-Chester Disease who have the BRAF V600 mutation, and melanoma in patients who have the BRAF V600E mutation.
**24**	DB07290	-7.8	Experimental
**25**	DB07321	-7.8	Experimental
**26**	DB01877	-7.8	Experimental
**27**	DB07685	-7.8	Experimental
**28**	DB04698	-7.8	Experimental
**29**	DB00414_Aceto	-7.8	Acetohexamide a sulfonylurea hypoglycemic agent that is metabolized in the liver to 1-hydrohexamide.

**30**	DB06629	-7.7	Investigational
**31**	DB14033	-7.7	Sulfisoxazole acetyl is an ester of sulfisoxazole, a broad-spectrum sulfanilamide and a synthetic analog of para-aminobenzoic acid (PABA) with antibacterial activity.
**32**	DB12378	-7.7	Investigational
**33**	DB16066	-7.7	Investigational
**34**	DB16103	-7.7	Investigational
**35**	DB08248	-7.7	Experimental
**36**	DB07312	-7.7	Experimental
**37**	DB02827	-7.7	Experimental
**38**	DB08942	-7.7	Isoxicam is a non-steroidal anti-inflammatory.
**39**	DB07270	-7.7	Experimental
**40**	DB02033	-7.7	Experimental
**41**	DB00278	-7.7	Argatroban is a synthetic direct thrombin inhibitor used for the prevention and treatment of thrombosis related to heparin use.
**42**	DB06999	-7.7	Experimental
**43**	DB07686	-7.7	Experimental
**44**	DB04140	-7.7	Experimental
**45**	DB00482	-7.7	Celecoxib is an NSAID used to treat osteoarthritis, rheumatoid arthritis, acute pain, menstrual symptoms, and to reduce polyps is familial adenomatous polyposis.
**46**	DB07000	-7.6	Experimental
**47**	DB12375	-7.6	Investigational
**48**	DB07136	-7.6	Experimental
**49**	DB11844	-7.6	Investigational
**50**	DB08162	-7.6	Investigational
**51**	DB07273	-7.6	Experimental
**52**	DB16078	-7.6	Investigational
**53**	DB11461	-7.6	Sulfachlorpyridazine is a sulfonamide antimicrobial used for urinary tract infections and in veterinary medicine.
**54**	DB13051	-7.6	Investigational
**55**	DB07556	-7.6	Experimental
**56**	DB12282	-7.6	Investigational
**57**	DB07369	-7.6	Experimental
**58**	DB07999	-7.6	Experimental
**59**	DB12068	-7.6	Investigational
**60**	DB07568	-7.5	Experimental
**61**	DB08560	-7.5	Experimental
**62**	DB12649	-7.5	Investigational
**63**	DB07964	-7.5	Experimental
**64**	DB16114	-7.5	Investigational
**65**	DB07534	-7.5	Experimental
**66**	DB07527	-7.5	Experimental
**67**	DB03202	-7.5	Experimental

**68**	DB14059	-7.5	Experimental
**69**	DB07605	-7.5	Experimental
**70**	DB08211	-7.5	Experimental
**71**	DB07414	-7.5	Experimental
**72**	DB07934	-7.5	Experimental
**73**	DB07522	-7.5	Experimental
**74**	DB00263	-7.5	Sulfisoxazole is a sulfonamide antibiotic used with other antibiotics to prevent and treat a variety of bacterial infections.
**75**	DB07540	-7.5	Experimental
**76**	DB08631	-7.5	Experimental
**77**	DB08000	-7.5	Experimental
**78**	DB06891	-7.5	Experimental
**79**	DB02479	-7.5	Experimental
**80**	DB07583	-7.5	Experimental
**81**	DB07748	-7.5	Experimental
**82**	DB04588	-7.5	Experimental
**83**	DB12399	-7.5	Investigational
**84**	DB00999	-7.4	Hydrochlorothiazide is a thiazide diuretic used to treat edema associated with a number of conditions, and hypertension.
**85**	DB01967	-7.4	Experimental
**86**	DB00695	-7.4	Furosemide is a loop diuretic used to treat hypertension and edema in congestive heart failure, liver cirrhosis, renal disease, and hypertension.
**87**	DB08170	-7.4	Experimental
**88**	DB13532	-7.4	Cyclopenthiazide is a thiazide diuretic with antihypertensive properties.
**89**	DB12476	-7.4	Investigational
**90**	DB07687	-7.4	Experimental
**91**	DB12921	-7.4	Investigational
**92**	DB06729	-7.4	Sulfaphenazole is a sulfonamide antibacterial.
**93**	DB09289	-7.4	Tianeptine is an atypical tricyclic antidepressant with antidepressant and anxiolytic effects primarily used to treat major depressive disorder.
**94**	DB14973	-7.4	Abrocitinib is a kinase inhibitor used to treat moderate-to-severe atopic dermatitis in adults.
**95**	DB08441	-7.4	Experimental
**96**	DB04244	-7.4	Experimental
**97**	DB02752	-7.4	Experimental
**98**	DB13284	-7.4	Meticrane is a diuretic medication. It has been marketed in Japan under the trade name Arresten and is used to lower blood pressure.
**99**	DB07220	-7.4	Experimental
**100**	DB06370	-7.4	Investigational
**101**	DB00808	-7.4	Indapamide is a thiazide diuretic used to treat hypertension as well as edema due to congestive heart failure.

**102**	DB00580	-7.4	Valdecoxib is a COX-2 inhibitor used to treat osteoarthritis and dysmenorrhoea.
**103**	DB07670	-7.4	Experimental
**104**	DB06835	-7.4	Experimental
**105**	DB15319	-7.4	Investigational
**106**	DB07572	-7.4	Experimental
**107**	DB08265	-7.4	Experimental
**108**	DB00839	-7.4	Investigational
**109**	DB15106	-7.4	Investigational
**110**	DB08527	-7.3	Experimental
**111**	DB03950	-7.3	Experimental
**112**	DB07724	-7.3	Experimental
**113**	DB07800	-7.3	Experimental
**114**	DB08487	-7.3	Experimental
**115**	DB13773	-7.3	Sulfamethoxypyridazine is a sulphonamide antibiotic indicated in the treatment of gonorrhea, inflammation, urinary tract ulcers, and bronchitis.
**116**	DB06140	-7.3	Investigational
**117**	DB02350	-7.3	Experimental
**118**	DB13675	-7.3	Experimental
**119**	DB04707	-7.3	Experimental
**120**	DB00436	-7.3	Bendroflumethiazide is a diuretic used to suppress lactation and to treat hypertension and edema.
**121**	DB15861	-7.3	Buthiazide is a diuretic indicated in the treatment of edema caused by congestive heart failure, as well as hepatic and renal diseases.
**122**	DB07927	-7.3	Experimental
**123**	DB08094	-7.3	Investigational
**124**	DB03526	-7.3	Experimental
**125**	DB13485	-7.3	Experimental
**126**	DB07732	-7.3	Experimental
**127**	DB01745	-7.3	Experimental
**128**	DB01120_Glic	-7.3	Gliclazide is a sulfonylurea used to treat hyperglycemia in patients with type 2 diabetes mellitus.
**129**	DB07323	-7.3	Experimental
**130**	DB11739	-7.3	Vonoprazan is a potassium-competitive acid blocker used in the treatment of acid-related disorders and as an adjunct to Helicobacter pylori eradication
**131**	DB07531	-7.3	Experimental
**132**	DB03656	-7.2	Experimental
**133**	DB04125	-7.2	Experimental
**134**	DB07252	-7.2	Experimental
**135**	DB15325	-7.2	Investigational
**136**	DB02917	-7.2	Experimental

**137**	DB01015	-7.2	Sulfamethoxazole is an oral sulfonamide antibiotic, given in combination with trimethoprim, used to treat a variety of infections of the urinary tract, respiratory system, and gastrointestinal tract.
**138**	DB08962	-7.2	Investigational
**139**	DB07730	-7.2	Experimental
**140**	DB07126	-7.2	Experimental
**141**	DB12574	-7.2	Investigational
**142**	DB00554	-7.2	Piroxicam is an NSAID used to treat the symptoms of osteoarthritis and rheumatoid arthritis.
**143**	DB07770	-7.2	Experimental
**144**	DB07688	-7.2	Experimental
**145**	DB02744	-7.2	Experimental
**146**	DB15316	-7.2	Investigational
**147**	DB12382	-7.2	Investigational
**148**	DB07947	-7.2	Experimental
**149**	DB11772	-7.2	Investigational
**150**	DB07746	-7.2	Experimental
**151**	DB07520	-7.2	Experimental
**152**	DB11462	-7.2	Sulfaethoxypyridazine: vet approved, has not been fully annoted.
**153**	DB11464	-7.2	Sulfaquinoxaline is a veterinary medicine which can be given to cattle and sheep to treat coccidiosis.
**154**	DB07322	-7.2	Experimental
**155**	DB13430	-7.2	Experimental
**156**	DB12254	-7.1	Investigational
**157**	DB02858	-7.1	Experimental
**158**	DB00880	-7.1	Chlorothiazide is a thiazide diuretic used to treat hypertension and edema in congestive heart failure, hepatic cirrhosis, and corticosteroid and estrogen therapy.
**159**	DB07692	-7.1	Experimental
**160**	DB05562	-7.1	Investigational
**161**	DB08645	-7.1	Experimental
**162**	DB06150	-7.1	Sulfadimethoxine is a sulfonamide antibiotic. Sulfadimethoxine is used to treat many infections, including treatment of respiratory, urinary tract, enteric, and soft tissue infections.
**163**	DB07309	-7.1	Experimental
**164**	DB07056	-7.1	Experimental
**165**	DB03818	-7.1	Experimental
**166**	DB16962	-7.1	Investigational
**167**	DB11992	-7.1	Investigational
**168**	DB01298	-7.1	Sulfacytine is a short-acting sulfonamide. The sulfonamides are synthetic bacteriostatic antibiotics with a wide spectrum against most gram-positive and many gram-negative organisms.
**169**	DB15097	-7.1	Investigational
**170**	DB07995	-7.1	Experimental

**171**	DB07876	-7.1	Experimental
**172**	DB03693	-7.1	Experimental
**173**	DB15024	-7.1	Investigational
**174**	DB08439	-7.1	Parecoxib is a selective COX-2 inhibitor and NSAID used for the short-term management of perioperative pain.
**175**	DB07541	-7.1	Experimental
**176**	DB01325	-7.1	Quinethazone, marketed as Hydromox, is a thiazide diuretic indicated for hypertension. Patients may experience adverse reactions such as dizziness, dry mouth, nausea, and hypokalemia.
**177**	DB12863	-7.1	Investigational
**178**	DB07848	-7.1	Experimental
**179**	DB08609	-7.1	Experimental
**180**	DB07944	-7.1	Experimental
**181**	DB00814	-7.1	Meloxicam is an NSAID used to treat osteoarthritis in adults, rheumatoid arthritis in adults, and juvenile rheumatoid arthritis in pediatrics.
**182**	DB16044	-7.1	Investigational
**183**	DB12022	-7.1	Investigational
**184**	DB04012	-7.1	Experimental
**185**	DB15630	-7.1	Investigational
**186**	DB08912	-7	Dabrafenib is a kinase inhibitor used to treat patients with specific types of melanoma, non-small cell lung cancer, and thyroid cancer.
**187**	DB00606	-7	As a diuretic, cyclothiazide inhibits active chloride reabsorption at the early distal tubule via the Na-Cl cotransporter, resulting in an increase in the excretion of sodium, chloride, and water.
**188**	DB01021	-7	Trichlormethiazide a thiazide diuretic with properties similar to those of hydrochlorothiazide.
**189**	DB08481	-7	Experimental
**190**	DB15250	-7	Investigational
**191**	DB07115	-7	Experimental
**192**	DB05943	-7	Investigational
**193**	DB08426	-7	Experimental
**194**	DB08213	-7	Experimental
**195**	DB13165	-7	Ripasudil is a rho kinase inhibitor indicated to treat ocular hypertension and open-angle glaucoma.
**196**	DB08495	-7	Experimental
**197**	DB13988	-7	Investigational
**198**	DB08132	-7	Experimental
**199**	DB07798	-7	Experimental
**200**	DB13803	-7	Experimental
**201**	DB08961	-7	Investigational
**202**	DB09215	-6.9	Droxicam is an oxicam non-steroidal anti-inflammatory drug and a prodrug of Piroxicam. It is used to reduce pain and inflammation in musculoskeletal disorders such as rheumatoid arthritis and osteoarthritis.

**204**	DB01124_Tol	-6.9	Tolbutamide is a sulfonylurea used to treat hyperglycemia in patients with type 2 diabetes mellitus.
**203**	DB00819_Aaz	-6.9	Acetazolamide is a carbonic anhydrase inhibitor used to treat edema from heart failure or medications, certain types of epilepsy, and glaucoma.

Figure S1. Alignment of proteins belonging to the α-family, headed by human isoform II. Active site amino acid residues are marked (*).

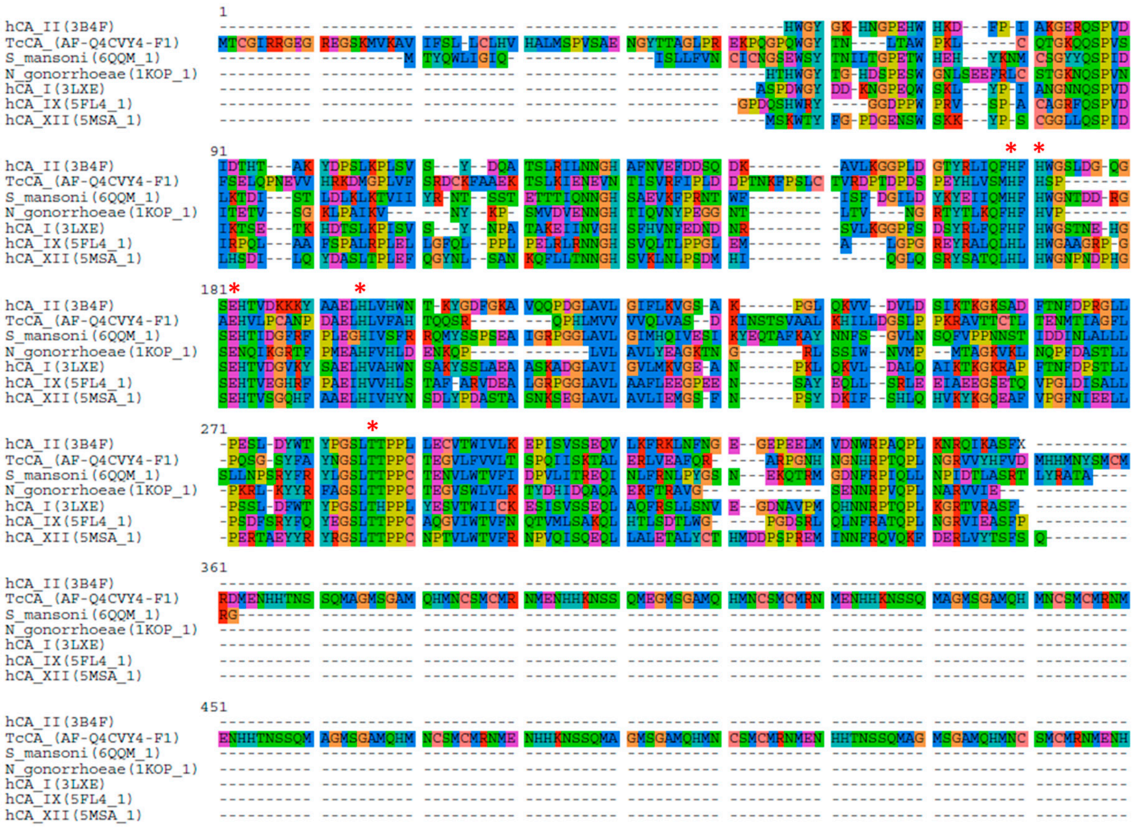

Figure S2. Interaction profile of the nine poses of acetazolamide (Aaz) from molecular docking with Gnina 1.0. Pose number one corresponds to the best binding free energy (6.9 Kcal/mol). Grey: hydrophobic interactions; cyan: hydrogen bond; orange: π-cation; purple: metallic bond.

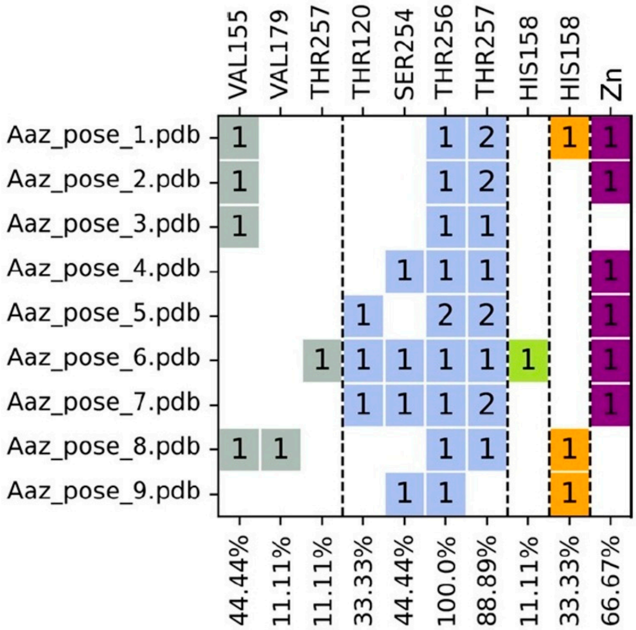

Figure S3. Interaction analysis of 45 candidate compounds in the active site of α- CA*Tc* of *T. cruzi.* Residues of interest in the active site are marked in the boxes (orange, green, yellow, light blue and purple).

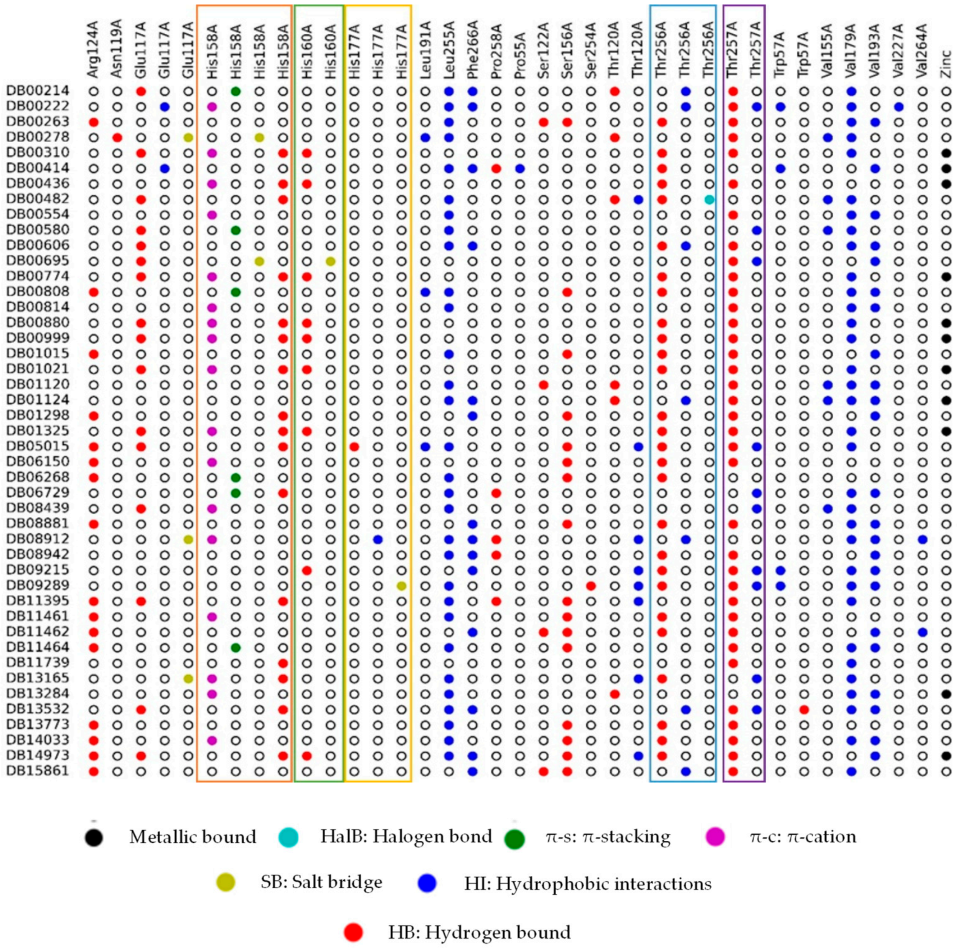

Supplement: Supplementary file 1 [file pharmaceuticals-18-00669-s001.zip › pharmaceuticals-3561029-supplementary.pdf]
